# Predicting the Effect of Adding a Citizenship Question to the 2020 Census

**DOI:** 10.1007/s13524-019-00803-4

**Published:** 2019-07-17

**Authors:** J. David Brown, Misty L. Heggeness, Suzanne M. Dorinski, Lawrence Warren, Moises Yi

**Affiliations:** 1 Center for Economic Studies, U.S. Census Bureau, 4600 Silver Hill Road, Washington, DC 20233 USA; 2 Research and Methodology Directorate, U.S. Census Bureau, 4600 Silver Hill Road, Washington, DC 20233 USA; 3 Social, Economic, and Housing Statistics Division, U.S. Census Bureau, 4600 Silver Hill Road, Washington, DC 20233 USA

**Keywords:** Citizenship, Immigration, Sensitive questions, Nonresponse, Administrative records

## Abstract

The addition of a citizenship question to the 2020 census could affect the self-response rate, a key driver of the cost and quality of a census. We find that citizenship question response patterns in the American Community Survey (ACS) suggest that it is a sensitive question when asked about administrative record noncitizens but not when asked about administrative record citizens. ACS respondents who were administrative record noncitizens in 2017 frequently choose to skip the question or answer that the person is a citizen. We predict the effect on self-response to the entire survey by comparing mail response rates in the 2010 ACS, which included a citizenship question, with those of the 2010 census, which did not have a citizenship question, among households in both surveys. We compare the actual ACS–census difference in response rates for households that may contain noncitizens (more sensitive to the question) with the difference for households containing only U.S. citizens. We estimate that the addition of a citizenship question will have an 8.0 percentage point larger effect on self-response rates in households that may have noncitizens relative to those with only U.S. citizens. Assuming that the citizenship question does not affect unit self-response in all-citizen households and applying the 8.0 percentage point drop to the 28.1 % of housing units potentially having at least one noncitizen would predict an overall 2.2 percentage point drop in self-response in the 2020 census, increasing costs and reducing the quality of the population count.

**Electronic supplementary material:**

The online version of this article (10.1007/s13524-019-00803-4) contains supplementary material, which is available to authorized users.

## Introduction

The self-response rate is a key driver of the cost and quality of a census. Nonresponding households are placed in nonresponse follow-up (NRFU), the most expensive census operation. In 2010, enumerators visited households up to six times trying to obtain an in-person interview. If unsuccessful, enumerators sought a proxy response from a neighbor or other knowledgeable individual. If no proxy response was received, the household count was imputed. Mule (2012) reported that the quality of proxy enumerations is significantly lower, on average, than that of self-response or in-person interviews, and imputations are likely to be of even lower quality.^1^

The addition of a citizenship question to the 2020 census could depress self-response rates, particularly for subpopulations such as noncitizens who are more sensitive to the question. The Census Act, Title 13 of the U.S. code, requires that responses to Census Bureau surveys and censuses be kept confidential and used only for statistical purposes (see Jarmin 2018). However, new survey evidence reported by McGeeney et al. (2019) suggests that some people fear that the Census Bureau will share their 2020 census answers with other government agencies and that the answers may be used against them.^2^ Such households could have confidentiality concerns regarding a citizenship question on the 2020 census questionnaire,^3^ and they may react by providing incorrect citizenship status, skipping the question, or not responding to the survey at all. Similarly, Escudero and Becerra (2018) reported that 75 % of men and 83 % of women in a survey in Providence, Rhode Island (the site of the 2018 End-To-End Census Test) agreed with the statement, “[M]any people in Providence County will be afraid to participate in the 2020 census because it will ask whether each person in the household is a citizen.” The self-response effect—when people choose not to respond to the survey—could be particularly damaging to census quality, affecting not only citizenship statistics but also other demographic statistics and the population coverage of the count itself. It could also significantly increase the cost of the 2020 census by requiring more NRFU.

Surveys asking respondents about participation in a future census are valuable for census planning but have important limitations. Respondent reports about whether they plan to respond in a future survey may not always align with subsequent behavior. Those expressing concern about a question in a focus group or an attitude survey may answer the same question in the actual census. Additionally, the respondent may predict the behavior of others even less reliably, and the questions are not designed to estimate the magnitude of self-response effects.

Our study instead investigates whether respondents in a survey containing the 2020 census citizenship question exhibited behavior consistent with having sensitivity about the question when asked to report the citizenship status of noncitizens in the household. By comparing mail response rates in the 2010 American Community Survey (ACS) (which contained the citizenship question) and the 2010 census (which did not) for the same housing units, we predict how adding the citizenship question to the 2020 census questionnaire could affect self-response rates. We focus on the differential effect on households that may contain noncitizens, given that they are more likely to have concerns about revealing citizenship status.

Our strategy for identifying a citizenship question effect is to conduct a difference-in-differences analysis comparing households likely to have concerns about the question with other households. We investigate the validity of this strategy by examining whether respondents displayed behavior consistent with the citizenship question being particularly sensitive when asked about a noncitizen in their household. Besides not self-responding, respondents could protect the noncitizen household member by skipping the question or providing an incorrect answer. To isolate the noncitizen effect from other factors, the difference-in-differences analysis compares item nonresponse or inconsistent response patterns for the citizenship question with those for the age question for noncitizens and citizens.

We would prefer to conduct a randomized controlled trial (RCT) in the current environment using two otherwise identical questionnaires: one containing a citizenship question and the other not.^4^ Unfortunately, the allowed timeframe prevents this. As of this writing, the Census Bureau is planning to conduct an RCT during the summer of 2019 as well as during the 2020 census within the Census Program for Evaluations and Experiments (CPEX), but those data will not be available before the 2020 census questionnaire is finalized. This study can serve as a benchmark for the RCTs from a period prior to the current public discourse about the citizenship question.

## Background

As discussed by Tourangeau and Yan (2007), the presence of a sensitive question on a questionnaire can lead to misreporting, item nonresponse, or unit nonresponse. Tourangeau and Yan argued that a question can be sensitive for multiple reasons. The question may be considered intrusive or an invasion of privacy. Such questions risk offending all respondents, regardless of their status on the question. Threat of disclosure (which we refer to as confidentiality concerns) raises fears that the information will be shared with others. The degree of respondent confidentiality concern may depend on whether answering truthfully will put them at risk. A special type of disclosure threat occurs when the question prompts socially undesirable answers. Tourangeau and Yan (2007) noted that the literature has found that respondents are more willing to report sensitive information in self-administered surveys than in interviewer-administered ones. If true, self-administered surveys could alleviate social desirability biases.

A few studies have estimated the effect of a sensitive question on unit response using RCTs. Dillman et al. (1993) analyzed data from an RCT in which one set of questionnaires included a question requesting the person’s Social Security number (SSN), and an alternative set excluded the SSN question. They found a 3.4 percentage point lower mail response rate for the questionnaires containing the SSN request. In areas with low mail response rates in the 1990 census, the difference was 6.2 percentage points. Similarly, Guarino et al. (2001) found a 2.1 percentage point lower self-response rate in high-response areas and a 2.7 percentage point lower rate in low-response areas in a 2000 census RCT with questionnaires including an SSN request than for questionnaires excluding such a request.

Foreign-born participants may engage in avoidance behavior when the survey includes a citizenship question. Camarota and Capizzano (2004) conducted focus groups with more than 50 field representatives for the Census 2000 Supplemental Survey (a pilot for the ACS). Field representatives reported that foreign-born respondents living in the country illegally or hailing from countries where there is distrust in government were less likely to participate. Some foreign-born respondents failed to list all household members. Field representatives suspected that some foreign-born respondents misreported citizenship status, and they believed this misreporting was due to “recall bias, a fear of the implications of certain responses or a desire to answer questions in a socially desirable way” (Camarota and Capizzano 2004).

Postcensus surveys asking about reasons for participation or nonparticipation in the census provide evidence about confidentiality concerns. Singer et al. (1993) reported that households with confidentiality concerns were less likely to self-respond to the 1990 census, and Singer et al. (2003) found that the belief that the census may be misused for law enforcement purposes was a significant negative predictor of self-response in the 2000 census. Even though Singer et al. (1993) hypothesized that foreign-born persons would have stronger confidentiality concerns due to concerns about immigration laws, their results showed no significant difference in concerns across foreign-born and native-born respondents.

O’Hare (2018) examined item response behavior to predict the effects of adding a citizenship question to the 2020 census. He found that the citizenship question has a higher item allocation rate (the sum of the item nonresponse and edit rates) in the ACS than other variables that will be included in the 2020 census. He also found that the citizenship item allocation rate is increasing over time and that it is higher for racial and ethnic minority groups, the foreign-born, and those self-responding. He concluded that these patterns support the idea that the citizenship question will affect self-response rates in the 2020 census, but he did not directly test it.

To our knowledge, our study is the first to estimate the effect of a citizenship question on self-response rates. It is also the first to examine item nonresponse and linked survey–administrative record (AR) reporting consistency patterns for citizenship status. We develop an alternative method for distinguishing self-response effects of sensitive questions when an RCT is unavailable.

## Data

We use the American Community Survey (ACS), the 2010 census, and ARs from the Social Security Administration (SSA) and the Internal Revenue Service (IRS).^5^ Our household survey sources come from the 2010 and 2017 ACS one-year files,^6^ 2005–2009 and 2012–2016 ACS five-year files, and the 2010 census. After the 2000 census, the Census Bureau’s principal citizenship data collection moved from the decennial long form to its replacement, the ACS. The ACS collects responses from approximately 1.6 % of households annually (U.S. Census Bureau 2016a, 2016b).^7^

**Fig. 1:**
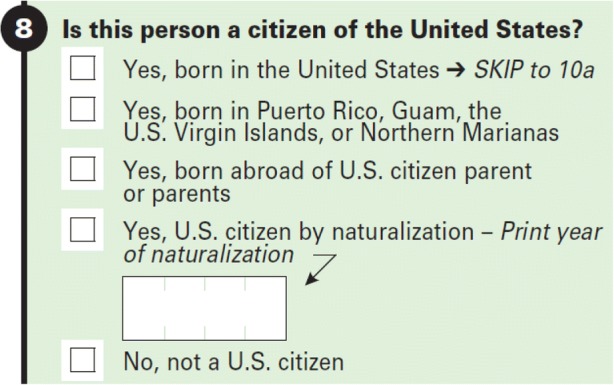
The 2010 American Community Survey (ACS) question on citizenship

As shown in Fig. 1, the citizenship question categorizes respondents as noncitizens or as citizens born in the United States, born in U.S. territories and Puerto Rico, born abroad to U.S. citizen parents, or of foreign nativity but naturalized. Our main AR source is the Census Numident, the most complete and reliable AR source of citizenship data currently available to the Census Bureau. The Numident is a record of individual applications for Social Security cards and certain subsequent transactions for those individuals. Unique, lifelong SSNs are assigned to individuals based on these applications. To obtain an SSN, the applicant must provide documented proof of citizenship status to the SSA.^8^

The SSA began requiring evidence of citizenship in 1972. Hence, citizenship data for more recently issued SSNs should be reliable as of the time of application.^9^ The SSA is not automatically notified when previously noncitizen SSN holders become naturalized citizens, so some naturalizations may be captured with a delay or not at all. To change citizenship status on an individual’s SSN card, naturalized citizens must apply for a new card, showing proof of the naturalization (U.S. passport or certificate of naturalization).^10^ Naturalized citizens wishing to work have an incentive to apply for a new card because noncitizen work permits expire, and the Numident is used in combination with U.S. Citizenship and Immigration Services (USCIS) data in the E-Verify program that confirms work eligibility of job applicants. Those with the strongest incentive to update their SSN card are job seekers or switchers, given that those employed in stable jobs may not be asked to reverify or update their status with a new valid SSN card immediately following naturalization.

The second AR source is Individual Taxpayer Identification Numbers (ITINs), issued by the IRS to those persons ineligible to obtain SSNs but who are required to file a federal individual income tax return. Persons with ITINs are noncitizens at the time of receipt of the ITIN by definition because all citizens are eligible to obtain SSNs.

We link SSN and ITIN records to the 2010 census and ACS data sets using a Protected Identification Key (PIK) developed by the Census Bureau.^11^ About 90.7 % of individuals in the 2010 census link to ARs, compared with 94.2 % in the 2010 ACS (see Luque and Bhaskar 2014; Rastogi and O’Hara 2012).^12^ Of those who matched, 57.6 million (20.6 % of linked persons) have missing citizenship data in the Numident, but the vast majority of these are U.S.-born.^13^ Although some noncitizen residents are assigned PIKs because they have ITINs, many without legal visas have neither SSNs nor ITINs and thus cannot be linked (see Bond et al. 2014). Given that residents without legal status may be especially sensitive to the citizenship question, our item nonresponse and ACS–AR disagreement analysis likely understates noncitizen sensitivity.

## Methods

### Item Response Methodology

To inform the design of our unit self-response analysis, we investigate whether households with noncitizens, in particular, exhibit behavior consistent with citizenship question sensitivity by examining citizenship question nonresponse among households that returned the questionnaire and the consistency of answers with ARs^14^ when the person being reported about (hereafter, *person of interest*) is an AR citizen versus an AR noncitizen. If only households containing noncitizens have concerns about the citizenship question, then we should see a higher incidence of problematic responses (skipping the question or providing an answer inconsistent with ARs) when respondents are asked about AR noncitizens, controlling for other relevant factors. This will help determine whether it is useful to compare all-citizen households with those potentially containing at least one noncitizen in our unit (household) self-response analysis.

Respondents could skip a question or provide an inconsistent response for other reasons, such as lack of knowledge regarding the person of interest’s characteristics or record linkage errors (the AR is for a different person) (see Tourangeau and Yan 2007). We control for these other reasons in several ways. First, we conduct the difference-in-differences analysis comparing a problematic response for the citizenship question with that of the age question for the same person of interest, separately for AR citizens and AR noncitizens. Problematic responses could occur for the same reasons for age and citizenship, with the exception that age responses are less likely to be related to citizenship question sensitivity. We classify age as being inconsistent in the survey and ARs if the values differ by more than one year.

Second, we control for other relevant factors that could explain differences in problematic responses to age and citizenship by estimating multivariate regressions with controls that proxy for such factors. Then, we conduct a Blinder-Oaxaca decomposition (Blinder 1973; Oaxaca 1973)^15^ of the differences between AR citizens and AR noncitizens into differences between the groups’ observed characteristics (explained portion) and other unobserved factors (unexplained portion). The explained portion includes differences in incidence across AR citizens and noncitizens of factors such as linguistic isolation, which may be associated with both citizenship status and a problematic response (via ability to understand the question). We attribute the unexplained portion to citizenship question sensitivity.

Before conducting the Blinder-Oaxaca decomposition, we estimate regressions for age and citizenship item nonresponse and age and citizenship status disagreement between the 2017 ACS and contemporaneous ARs. The regressions are of the following form:

1 $$ {Y}_{G_j AGE}={\mathbf{X}}_{G_j}^{\prime }{\upbeta}_{G_j AGE}+{\upvarepsilon}_{G_j AGE}. $$

2 $$ {Y}_{G_j CITIZENSHIP}={\mathbf{X}}_{G_j}^{\prime }{\upbeta}_{G_j CITIZENSHIP}+{\upvarepsilon}_{G_j CITIZENSHIP}. $$

Person of interest *j* belongs to one of two groups *G* ∈ (*N*, *C*), where the *N* group (AR noncitizens) could be harmed by confidentiality breaches regarding a citizenship question or are otherwise sensitive to the question, while the *C* group (AR citizens) could not be. Eqs. (1) and (2) are estimated separately for the *N* and *C* groups. *Y* is the dependent variable for person *j* in group *G*, **X** is a vector of characteristics, β contains the slope parameters and intercept, and ε is a regression error term with a conditional mean of 0, given **X**.

In the item nonresponse regressions, *Y* is equal to 1 if there is no response for the question for person of interest *j* in group *G*, and 0 otherwise (even if the response was later edited or allocated). In the ACS–AR age disagreement regressions, *Y* is equal to 1 if the difference in age across sources is more than one year, and 0 otherwise. Persons who have age in AR data and reported age in the 2017 ACS are included in these regressions. For the ACS–AR citizenship disagreement regressions, *Y* is equal to 1 if the two sources indicate different citizenship statuses, and 0 if both sources agree. Persons who have AR citizenship and reported citizenship in the 2017 ACS are included in the citizenship disagreement regressions.

The **X** variables include person of interest *j*’s relationship to the reference person,^16^ working in the last week, searching for a job in the last four weeks, race/ethnicity, and an indicator for better- or worse-quality person linkage;^17^ reference person sex and educational attainment (less than high school, high school but less than bachelor’s degree, bachelor’s degree, and graduate degree); six household income categories; a household linguistic isolation indicator with three categories, including linguistically isolated households (no person 14 years or older speaks only English or reports speaking it “very well”), not linguistically isolated households (at least one person 14 years or older speaks another language at home, and at least one person 14 years or older speaks only English or reports speaking it “very well”), and only English (all persons 14 years and older speak only English at home); an indicator for self-response (equal to 1 for mail or Internet response, and 0 for in-person or telephone interview); share of households by block group with at least one noncitizen in the 2012–2016 five-year ACS; and share of households below the poverty level by block group in the 2012–2016 five-year ACS.

Relationship may proxy for the amount of knowledge the reference person has about the person of interest. If so, less item nonresponse and disagreement would be expected when respondents report about themselves than about others, especially nonrelatives.^18^ Alternatively, respondents may feel they have less right to disclose sensitive information about others. Social desirability could also lead to discrepancies with administrative data, and it is likely to be more of a factor when respondents report about themselves

Linguistic isolation could be associated with misunderstandings from translation or interpretation, leading to item nonresponse and inconsistent reporting.^19^ It could also proxy for how well the household is integrated into U.S. society. Households that are less well integrated may have less understanding about the survey, for example, leading to a less complete and accurate response. Reference person education and household income may also be associated with question comprehension. Reference person sex and person of interest race/ethnicity may be associated with different sensitivity to questions not specific to citizenship. Person of interest labor market activity could be associated with greater reference person knowledge about the person of interest’s citizenship status because the status may affect the person’s employment eligibility. Record linkage errors could cause inconsistent reporting because the AR and ACS persons would be different.

As mentioned, Tourangeau and Yan (2007) reported that studies have found less item nonresponse and inconsistent reporting about sensitive questions in self-responses (as opposed to interviewer-administered surveys), consistent with social desirability being a factor in interviews. McGovern (2004), however, reported item allocation rates for citizenship and other related questions that are twice as high in mail responses compared with telephone or personal interviews in the ACS.

Neighborhood shares of households below the poverty line or with noncitizens could be associated with different levels of openness on government surveys.

For the Blinder-Oaxaca decomposition, we create summary measures of problematic response to the age and citizenship questions. Each variable is set to 1 if the respondent does not provide a response to the question, the respondent’s answer is edited,^20^ or the answer is inconsistent with ARs; and it is 0 if an answer is provided that is consistent with ARs. Cases in which ARs are missing are excluded. We set the problematic-response dependent variables ${Y}_{G_j AGE}$ and ${Y}_{G_i CITIZENSHIP}$ equal to 1 if the response regarding person of interest *j* in group *G* is problematic for the age and citizenship questions, respectively, and 0 otherwise.^21^ The difference between the responses is

3 $$ \Delta  {Y}_{G_j}={Y}_{G_j CITIZENSHIP}-{Y}_{G_j AGE}. $$

We estimate regression models for each group:

4 $$ \Delta  {Y}_{N_j}={\mathbf{X}}_{N_j}^{\prime }{\upbeta}_N+{\upvarepsilon}_{N_j}. $$

5 $$ \Delta  {Y}_{C_j}={\mathbf{X}}_{C_j}^{\prime }{\upbeta}_C+{\upvarepsilon}_{C_j}. $$

The difference-in-differences in expected problematic response rates across the two questions for the two groups *NC* and *C* is

6 $$ \Delta  \Delta  {Y}_{NC}=E\left({\Delta  Y}_N\right)-E\left({\Delta  Y}_C\right). $$

We decompose this as follows:

7 $$ \Delta  \Delta  {Y}_{NC}={\left[E\left({\mathbf{X}}_N\right)-E\left({\mathbf{X}}_C\right)\right]}^{\prime }{\upbeta}_C+\left[E{\left({\mathbf{X}}_{NC}\right)}^{\prime}\left({\upbeta}_{NC}-{\upbeta}_C\right)\right]. $$

The first term (explained variation) applies the coefficients for the AR citizen group to the difference between the expected value of the AR noncitizen group’s predictors and those of the AR citizen group. The second (unexplained variation) is the difference between the expected value of the AR noncitizen group’s predictors applied to the AR noncitizen group’s coefficients and the same predictors applied to the AR citizen group’s coefficients. The interpretation that the unexplained variation represents the variation due to the AR citizenship status of the person of interest is dependent on the assumption that there are no unobserved variables relevant to the difference-in-differences in problematic response across the two questions and AR citizenship groups.

### Housing Unit Self-response Methodology

There are several elements to our method for predicting the effect of adding a citizenship question to the 2020 census on housing unit self-response rates. We take advantage of a natural experiment setting. In 2010, a subset of housing units that responded to the census were randomly selected to also participate in the 2010 ACS using a probability sampling scheme that did not depend on the citizenship status of individuals in the selected households. The ACS questionnaire contained 75 questions, including a battery of three questions that asked about nativity, citizenship status, and year of immigration. These same households also received a list of 10 questions from the full-count census questionnaire that did not include citizenship. Both the ACS and the census are mandatory Title 13 surveys that households are required by law to complete. We focus on census housing units^22^ that received both questionnaires by mail from the initial mailing, did not have the questionnaire returned as undeliverable as addressed by the U.S. Postal Service, and were not classified as a vacant or delete (meaning unoccupied, uninhabitable, or nonexistent). We define a 2010 census self-response as a returned questionnaire from the first mailing that is not blank. For the 2010 ACS, a self-response is a mail response, also from the first contact mailing.

The simple difference in self-response rates (mail response) between the two surveys does not control for other reasons a household might respond to one survey and not the other besides the presence/absence of a citizenship question. Census self-response is bolstered by a media campaign and intensive community advocacy group support, and the ACS questionnaire involves much greater respondent burden (Office of Management and Budget 2008, 2009).^23^

We control for the effects of other factors on the difference between ACS and census self-response rates by comparing the difference in households likely to have concerns about the citizenship question with the difference in households unlikely to have such concerns. AR noncitizens could be put at risk if their personal information regarding citizenship status and location were shared with immigration enforcement agencies, but AR citizens would not be put at risk. Households containing at least one noncitizen may thus have concerns about participating in a survey specifically containing a citizenship question, but all-citizen households presumably do not have such concerns. Our analysis assumes that any reduction in self-response to the ACS versus the census for all-citizen households is due to factors other than the presence of a citizenship question.

In our dichotomy, the less-sensitive group is “all-citizen households,” those households where all persons reported in the ACS to be living in the household at the time of the survey are AR citizens, and all are reported citizens in the ACS as well. The more sensitive group, “other households,” includes those households where (1) some residents may be both AR citizens and as-reported citizens but at least one resident is not; (2) there is disagreement between the survey report and AR response; or (3) citizenship status is not reported in one or both sources. This expands the group of people potentially having citizenship question confidentiality concerns compared with those we are using in the problematic response analysis. AR noncitizens are probably not the people most sensitive to a citizenship question, given that most of them are legal residents. Because we are unable to distinguish undocumented residents without SSNs or ITINs from citizens or noncitizen legal residents with SSNs or ITINs but have personally identifiable information discrepancies that prevent a link to ARs, we include all persons with missing AR citizenship in the sensitive group here. We use the ACS household roster to define which people are living in the household.

We assume that all-citizen households are less sensitive to the citizenship question than other households because, as we show, respondents have demonstrated a willingness to provide citizenship status answers for AR citizens, and those answers are quite consistent with ARs and thus are likely truthful responses. In comparison with others, more of the all-citizen household group’s reluctance to self-respond to the ACS should be due to reasons other than the citizenship question, such as unwillingness to answer a longer questionnaire. Note that if some of the reluctance by all-citizen households to self-respond is due to the citizenship question in the ACS, that will downwardly bias our estimate of the citizenship question unit self-response effect.^24^

A different magnitude for the decline in self-response rates for the other household group relative to all-citizen households may not actually be due to greater sensitivity. Other characteristics besides citizenship status could be associated with different ACS self-response, and the two household groups could have different propensities to have such characteristics. To control for this possibility, we perform Blinder-Oaxaca decompositions to isolate citizenship question concerns. We use multiple methods for the Blinder-Oaxaca decomposition. The traditional method of relying on the literature to model factors related to observed characteristics that may drive self-response is reported as our main findings. Robust models using lasso and principal components techniques to identify the main observable factors explaining variation are included in the online appendix.

In our model, households belong to one of two groups *G* ∈ (*S*, *U*), where the *S* group is thought to be potentially sensitive to a citizenship question (other households), and the *U* group is not (all-citizen households). We set the self-responses ${R}_{G_i{ACS}_t}$ and ${R}_{G_i{Census}_t}$ equal to 1 if household *i* in group *G* self-responds in year *t* to the ACS and census, respectively, and 0 otherwise.^25^ The difference between the survey responses is

8 $$ \Delta  {R}_{G_it}={R}_{G_i{ACS}_t}-{R}_{G_i{Census}_t}. $$

Our choice for the vector of predictors **X** draws from Erdman and Bates (2017), who developed a block group–level model to predict census self-response rates.^26^ Factors that predict census self-response may be even more important for a more burdensome questionnaire. We use household-level or household reference person equivalents for their variables:^27^ log household size and its square, owned versus other, housing structure type (single-unit structure, multiunit, and other), household income, presence of children (related under 5, related 5–17, unrelated under 5, and unrelated 5–17), presence of an unrelated adult, all adults worked in the last week, reference person characteristics (married male, married female, unmarried male, unmarried female, race/ethnicity, age categories, educational attainment, moved here two to five years ago, and moved here within the last year), tract population density in the 2010 census,^28^ and the shares of housing units in the block group that are vacant and under the poverty level. We add indicators for linguistically isolated households and not linguistically isolated households given McGovern’s (2004) finding that linguistically isolated households self-respond to the ACS at lower rates than only English-speaking households. Because immigrants tend to be concentrated in particular neighborhoods^29^ and such neighborhoods are more exposed to community outreach encouraging census response (see U.S. Census Bureau 2019),^30^ we also control for the block group–level share of housing units with at least one noncitizen.

We estimate regression models for each household group where β contains the slope parameters and intercept, and ε is a regression error term with conditional mean of 0, given **X**.

9 $$ \Delta  {R}_{S_{it}}={\mathbf{X}}_{S_{it}}^{\prime }{\upbeta}_{S_t}+{\upvarepsilon}_{S_{it}.} $$

10 $$ \Delta  {R}_{U_{it}}={\mathbf{X}}_{U_{it}}^{\prime }{\upbeta}_{U_t}+{\upvarepsilon}_{U_{it}}. $$

The difference-in-differences in expected self-response rates across the two surveys for the two groups *S* and *U* in year *t* is

11 $$ \Delta  \Delta  {R}_{SU_t}=E\left({\Delta  R}_{S_t}\right)-E\left({\Delta  R}_{U_t}\right). $$

We decompose this as follows:

12 $$ \Delta  \Delta  {R}_{SU_t}={\left[E\left({\mathbf{X}}_{S_t}\right)-E\left({\mathbf{X}}_{U_t}\right)\right]}^{\prime }{\upbeta}_{U_t}+\left[E{\left({\mathbf{X}}_{S_t}\right)}^{\prime}\left({\upbeta}_{S_t}-{\upbeta}_{U_t}\right)\right]. $$

The first term (explained variation) applies the coefficients for the unsensitive group to the difference between the expected value of the sensitive group’s predictors and those of the less-sensitive group. The second (unexplained variation) is the difference between the expected value of the sensitive group’s predictors applied to the sensitive group’s coefficients and the same predictors applied to the unsensitive group’s coefficients. The interpretation that the unexplained variation represents the citizenship question effect is dependent on the assumption that there are no unmeasured confounding variables relevant to the difference-in-differences in self-response across the two surveys.

To study how changes in predictors over time might affect the magnitude of the unexplained variation (*UV*) in the decomposition, we apply the coefficients from the 2010 models to the predictors as measured in the 2017 ACS:^31^

13 $$ {UV}_{2017}=E{\left({\mathbf{X}}_{S_{2017}}\right)}^{\prime }{\upbeta}_{S_{2010}}-E{\left({\mathbf{X}}_{S_{2017}}\right)}^{\prime }{\upbeta}_{U_{2010}}. $$

## Analysis^32^

### Problematic Response

Table 1 reports item (question) nonresponse rates for age and citizenship in the 2017 ACS and age and citizenship status disagreement rates between the 2017 ACS and 2017 ARs, separately for AR citizens and noncitizens. Item nonresponse is very low for age, and it is only approximately 0.5 percentage points higher for AR noncitizens than citizens. The item nonresponse rate for citizenship is actually lower than the rate for age among AR citizens, but it is 4 percentage points higher for AR noncitizens. The disagreement rates for both questions are higher for AR noncitizens, which could partly reflect less knowledge and understanding of the questions from noncitizens. The gap between AR noncitizens and citizens is much larger for the citizenship question. AR citizens have age discrepancies 10 times more often than citizenship discrepancies, whereas AR noncitizens have citizenship discrepancies 6 times more often than age discrepancies. The citizenship disagreement rate is 92 times larger for AR noncitizens than for AR citizens (39.7 % vs. 0.4 %).

**Table 1:** Summary statistics for ACS item nonresponse and AR–ACS disagreement regressions

	AR Citizens			AR Noncitizens		
Variable	Mean	SE	Sample Size	Mean	SE	Sample Size
Age Item Nonresponse	0.85	0.008	4,108,000	1.32	0.03	253,000
Citizenship Item Nonresponse	0.44	0.005	4,108,000	4.42	0.07	253,000
ACS–AR Age Disagreement	4.58	0.02	4,060,000	6.42	0.07	249,000
ACS–AR Citizenship Disagreement	0.43	0.006	3,872,000	39.73	0.14	229,000

*Notes:* The sample sizes are unweighted, and the means and standard errors are survey-weighted. The standard errors are calculated using Fay’s balanced repeated replication variance estimation method, with 80 replicate weights, adjusting the original weights by a coefficient of 0.5. Group quarters and Puerto Rico are excluded from the sample.

*Source:* American Community Survey (ACS), Census Numident, and ITINs, 2017. The Disclosure Review Board release number is DRB-B0035-CED-20190322.

Next, we attempt to distinguish the extent to which the differences in Table 1 can be attributed to citizenship question concerns by AR noncitizens versus other factors correlated with both citizenship status and response behavior. Table 2 shows results from multivariate regressions predicting age and citizenship item response and ACS–AR disagreement separately for persons who are AR citizens and noncitizens. The coefficients for AR noncitizens in the estimated equations for citizenship item nonresponse and ACS–AR disagreement are very different from the comparable regression coefficients for age, regardless of AR citizenship status or for citizenship of AR citizens. Age item nonresponse and ACS–AR disagreement are greater when the person of interest is a nonrelative, suggesting that lack of knowledge is a contributing factor. This is also true for citizenship item nonresponse of AR noncitizens, but citizenship disagreement is actually greater when reporting about oneself; thus, confidentiality concerns may be playing a role for the citizenship question for AR noncitizens.

**Table 2:** Item nonresponse and ACS–AR disagreement regressions

	Age Item Nonresponse		Citizenship Item Nonresponse		ACS–AR Age Disagreement		ACS–AR Citizenship Disagreement	
	AR Citizen	AR Noncitizen	AR Citizen	AR Noncitizen	AR Citizen	AR Noncitizen	AR Citizen	AR Noncitizen
Relative	0.116	0.188	–0.382	–1.105	0.000	0.010	–0.069	–7.080
	(0.013)	(0.061)	(0.010)	(0.087)	(0.000)	(0.001)	(0.010)	(0.248)
Nonrelative	2.552	3.057	1.093	4.839	0.036	0.058	0.499	–15.987
	(0.086)	(0.299)	(0.053)	(0.370)	(0.001)	(0.005)	(0.054)	(0.731)
Non-Hispanic African American	0.372	0.077	0.393	2.515	0.015	–0.022	0.024	1.660
	(0.032)	(0.162)	(0.016)	(0.185)	(0.001)	(0.003)	(0.015)	(0.661)
Hispanic	–0.065	–0.450	0.030	3.738	0.014	0.014	0.620	–4.817
	(0.033)	(0.111)	(0.026)	(0.156)	(0.001)	(0.002)	(0.030)	(0.483)
Other Non-Hispanic	0.108	–0.024	0.856	2.304	0.039	0.016	0.536	–0.459
	(0.037)	(0.102)	(0.035)	(0.141)	(0.001)	(0.002)	(0.035)	(0.450)
Not Linguistically Isolated	0.118	–0.348	1.412	1.810	0.007	–0.113	0.924	–8.477
	(0.030)	(0.119)	(0.026)	(0.136)	(0.001)	(0.003)	(0.026)	(0.478)
Linguistically Isolated	0.191	–0.084	1.825	1.049	0.021	–0.126	5.187	–16.86
	(0.074)	(0.148)	(0.073)	(0.177)	(0.002)	(0.003)	(0.149)	(0.561)
Better Linkage	–0.932	–1.658	–0.079	–0.509	–0.000	–0.006	–0.767	3.533
	(0.033)	(0.125)	(0.015)	(0.154)	(0.001)	(0.002)	(0.024)	(0.397)
Mail/Internet Response	–0.304	–1.035	0.416	5.280	–0.008	–0.020	–0.100	3.672
	(0.023)	(0.085)	(0.013)	(0.133)	(0.001)	(0.002)	(0.013)	(0.346)
Weighted Observations	262,800,000	20,700,000	262,800,000	20,700,000	259,500,000	20,350,000	248,400,000	19,030,000
Unweighted Observations	4,108,000	253,000	4,108,000	253,000	4,060,000	249,000	3,872,000	229,000

*Notes:* These regressions are estimated by ordinary least squares (OLS), weighted by ACS person weights. Standard errors, shown in parentheses, are clustered by block group. We also include person of interest worked in the last week and searched for a job within the last four weeks; reference person sex and educational attainment (less than high school, high school but less than bachelor’s degree, bachelor’s degree, and graduate degree); household income categories; and share of households in the block group that contain at least one noncitizen and the share of households in the block group below the poverty level. Group quarters and Puerto Rico are excluded from the sample.

*Source:* American Community Survey (ACS), Census Numident, and ITINs, 2017. The Disclosure Review Board release number is CBDRB-FY19-CMS-7917.

Misunderstandings due to language barriers can help explain age and citizenship disagreement for AR citizens, whereas English-only households have more citizenship disagreement for AR noncitizens. Record linkage errors may explain some of the age disagreement for AR noncitizens and the citizenship disagreement for AR citizens, but they do not explain the citizenship disagreement for AR noncitizens. Self-response is associated with lower nonresponse rates and lower ACS–AR disagreement rates for age. It is also associated with lower citizenship disagreement rates for AR citizens. This may reflect greater cooperation among self-responders. Self-responders have higher nonresponse to the citizenship question, however, and there is more disagreement on citizenship for AR noncitizens. It is possible that field representatives are able to allay respondent confidentiality concerns.^33^ This result is inconsistent with social desirability, which should lead to higher nonresponse and disagreement for sensitive questions in personal interviews. The Hispanic-origin effects are very different for AR citizens and noncitizens: Hispanic AR noncitizen respondents are more likely to skip the citizenship question, but they are less likely to give a discrepant answer.

In sum, these results suggest that the very different citizenship question response behavior when asked about AR noncitizens is associated with citizenship question sensitivity, not lack of knowledge, misunderstandings, or record linkage errors. Among the reasons for sensitivity, the results are most consistent with confidentiality concerns, which are particularly relevant for unit self-response. Those who are legally vulnerable may have confidentiality concerns about all their data and thus may not participate at all.

To more rigorously distinguish how much of the response difference for the citizenship question when asked about AR noncitizens is due to the AR noncitizen status itself versus other factors correlated with response behavior and AR citizenship status, we perform a Blinder-Oaxaca decomposition of differences in problematic response to the citizenship and age questions (Eq. (7)); results are shown in Table 3. AR citizens have virtually no difference in the problematic response rate across the two questions, but that rate is 36.6 percentage points higher for citizenship when the person of interest is an AR noncitizen. None of this gap can be explained by differences in observable characteristics between AR citizens and noncitizens. In fact, the distribution of characteristics for response about AR citizens is more strongly associated with problematic citizenship response than that for response about AR noncitizens. These results suggest that respondents are particularly sensitive about providing citizenship status for AR noncitizens. This motivates our use of household members’ citizenship status to divide households into ones more likely to be sensitive to the citizenship question versus those less likely to be sensitive in the housing unit self-response analysis presented in the next section.

**Table 3:** Blinder-Oaxaca decomposition of the differences in problematic response to the citizenship and age questions by AR citizenship status

	Problematic Response Rate (%)		
	Citizenship	Age	Difference
AR Noncitizens	44.6	8.0	36.6
	(0.15)	(0.07)	(0.17)
AR Citizens	5.9	5.8	0.1
	(0.03)	(0.02)	(0.04)
Difference-in-Differences			36.5
			(0.08)
Explained			–1.0
			(0.04)
Unexplained			37.4
			(0.09)

*Notes:* The results use ACS person weights. The sample excludes observations where age or citizenship is missing from AR. The response is problematic if no answer is provided about the item, the answer is changed in the edit process, or the answer is inconsistent with the AR record for the person. The response is not problematic if the answer is consistent with the person’s AR record. Standard errors are shown in parentheses. The standard errors for the differences are bootstrapped using 80 ACS replicate weights. The number of observations is 4,361,000.

*Source:* ACS one-year file, Census Numident, and ITINs, 2017. The Disclosure Review Board release number is DRB-B0035-CED-20190322.

### Effect of the Citizenship Question on Housing Unit Self-response Rates

We now forecast the effect of adding a citizenship question to the 2020 census on housing unit self-response rates by comparing mail response rates in the 2010 census and the 2010 ACS for the same housing units, separately for all-citizen households according to both the ACS and AR versus households potentially containing at least one noncitizen (other households) (Eq. (12)).

Table 4 displays the Blinder-Oaxaca decomposition. The self-response rate is higher in the 2010 census than the ACS for both household categories, presumably reflecting the higher burden and limited marketing strategy of the ACS. The all-citizen self-response rate is greater than the other household rate in each survey, suggesting that other households have a lower self-response rate in general. Most important for this study is understanding how the difference in housing unit self-response rate across groups varies between the 2010 census and ACS. Although the self-response rate for all-citizen households is 8.9 percentage points lower in the ACS than in the 2010 census, the self-response rate for households potentially containing at least one noncitizen is 20.7 percentage points lower for the ACS than the self-response rate to the 2010 census, which is a 11.9 percentage point difference between the two categories. Of this difference, 8.8 percentage points are unexplained.^34^

**Table 4:** Blinder-Oaxaca decomposition of the differences in 2010 ACS to 2010 census self-response rates by household citizenship type

	Self-response Rate (%)		
	2010 ACS	2010 Census	Difference
All Other Households	42.0	62.7	–20.7
	(0.32)	(0.14)	(0.12)
AR and ACS All-Citizen Households	65.6	74.4	–8.9
	(0.33)	(0.11)	(0.12)
Difference-in-Differences			–11.9
			(0.07)
Explained			–3.1
			(0.08)
Unexplained			–8.8
			(0.11)

*Notes:* Only NRFU–eligible housing units are included. 2010 CUF self-response is nonblank response to the first mailing, and ACS self-response is mail response. The standard errors are shown in parentheses, and they are bootstrapped using 80 ACS replicate weights. The number of observations is 1,418,000.

*Source:* ACS one-year file, Census Unedited File (CUF), Census Numident, and ITINs, 2010. The Disclosure Review Board release number is DRB-B0035-CED-20190322.

Because the characteristics of households in the two categories change over time and we want to make the most up-to-date prediction possible, we apply the 2010 model coefficients to 2017 ACS characteristics in Table 5 (Eq. (12)). The unexplained portion declines slightly to 8.0 percentage points. We consider this our best estimate of the effect of the citizenship question on unit self-response in households potentially containing at least one noncitizen.

**Table 5:** Predicted 2017 ACS to 2010 census response rate differences for other households using other household versus all-citizen models

Model	2017 ACS – 2010 Census
All Other Household Model	–19.9
	(0.40)
AR and ACS All-Citizen Household Model	–11.9
	(0.31)
Difference-in-Differences	–8.0
	(0.51)

*Notes:* Only NRFU-eligible housing units are included. 2010 census self-response is nonblank response to the first mailing, and ACS self-response is mail response. The standard errors are shown in parentheses. The standard errors for the 2017 ACS – 2010 census response differences are calculated using Fay’s balanced repeated replication variance estimation method, with 80 replicate weights, adjusting the original weights by a coefficient of 0.5. The difference-in-differences (*DiD*) standard errors (*SE*) are calculated as $DiD\  SE=\sqrt{SE{\left({Est}_1\right)}^2+ SE{\left({Est}_2\right)}^2}$, where the two estimates (*Est*) are the 2017 ACS – 2010 census differences for the two groups. They are the standard errors of the model predictions, based on the bootstrapped regressions in Eqs. (9) and (10) that use 80 ACS replicate weights. The estimates use ACS housing unit weights. The all other households group makes up 28.1 % of housing units in 2017. The number of observations is 755,000.

*Source:* ACS one-year file, Census Numident, and ITINs, 2017. The Disclosure Review Board release number is DRB-B0035-CED-20190322.

We note three caveats to this analysis. First, it assumes that the self-response rate of all-citizen households will be unaffected by the addition of a citizenship question. Some all-citizen households could boycott the census in solidarity with noncitizens, whereas others may become more excited to participate, and it is unclear which effect will be larger or whether they will cancel each other out. Second, the group of households potentially containing at least one noncitizen most likely includes some all-citizen households, but we are unable to distinguish them because of incomplete citizenship coverage in the ACS and administrative data (and in linkage between them) as well as disagreement across sources. Including some all-citizen households in this group may understate the citizenship question effect on households actually containing at least one noncitizen. Third, this analysis does not capture changes over time in the degree of sensitivity to a citizenship question (e.g., due to changes in policy, trust in government, or public discourse about the question) for a housing unit with a fixed set of characteristics. That would require estimating models on fresher surveys with and without a citizenship question. Planned RCTs in the summer of 2019 and in 2020 can do this.

## Conclusion

Our study finds that respondents often provide answers to the citizenship question that conflict with ARs or skip the question altogether when asked about AR noncitizens, raising concerns about the quality of survey-sourced citizenship data for the noncitizen subpopulation. This happens much less frequently when asked about AR citizens’ citizenship status or when asked about either AR citizens’ or noncitizens’ age. Lack of knowledge about the person of interest’s citizenship status, misunderstanding the question, record linkage errors, and social desirability concerns do a poor job of explaining these patterns. After controlling for alternative explanations for such behavior, we still find that problematic reactions are much more frequent when respondents are asked about the citizenship status of AR noncitizens. We interpret this as evidence that respondents have citizenship question sensitivity that may be due to confidentiality concerns or concerns about inappropriate statistical use of the data regarding AR noncitizens, who are more legally vulnerable to these misuses.

We take advantage of a natural experiment in which a scientific probability sample of housing unit addresses were in both the 2010 ACS, which contained a citizenship question, and the 2010 census, which did not include the question. We compare the difference in ACS and census self-response in households likely to be sensitive to the citizenship question (those potentially containing at least one noncitizen) versus those unlikely to be sensitive to it (all-citizen households), and we find an 8.8 percentage point larger drop in self-response rates in the ACS versus the census in households potentially containing at least one noncitizen. When the 2010 coefficients are applied to 2017 ACS characteristics, the estimate declines slightly to 8.0 percentage points. Assuming that the citizenship question does not affect unit self-response in all-citizen households and applying the 8.0 percentage point drop to the 28.1 % of housing units potentially having at least one noncitizen estimates an overall 2.2 percentage point drop in housing unit self-response in the 2020 census. This would result in more NRFU fieldwork, more proxy responses, and a lower-quality population count.

## Electronic supplementary material

ESM 1 (DOCX 17 kb)

## Footnotes

1 Using the 2010 Census Coverage Measurement Survey, Mule (2012) found correct enumeration rates of 97.3 % for mail-back responses and 70.2 % for proxy responses. Rastogi and O’Hara (2012) reported person linkage rates between the 2010 census and administrative records of 96.7 % for mail-back responses and 33.8 % for proxy responses, indicating that the completeness and accuracy of personally identifiable information in proxy responses is poor.

2 In the 2020 Census Barriers, Attitudes, and Motivators Study (CBAMS), 32.5 % of foreign-born survey respondents reported being “extremely concerned” or “very concerned” that the Census Bureau will share their answers with other government agencies, and 34.0 % were “extremely concerned” or “very concerned” that their answers will be used against them. This compares with overall rates of 24.0 % and 22.0 %, respectively (McGeeney et al. 2019). One CBAMS focus group participant said, “Every single scrap of information that the government gets goes to every single intelligence agency, that’s how it works . . . individual-level data. Like, the city government gets information and then the FBI and then the CIA and then ICE and military” (Evans et al. 2019:42).

3 Evans et al. (2019) reported that some CBAMS focus group participants said the purpose of the citizenship question is to find undocumented immigrants. One said, “[The question is used] to make people panic. Some people will panic because they are afraid that they might be deported” (p. 59).

4 Respondent behavior may be different in the 2020 census than in 2010 because of changes in policy, public trust in government, and public discourse about the citizenship question.

5 We have restricted access to these data under an approved Census Bureau project. Other researchers may develop a project proposal and request restricted access to similar data for replication and further study. Researchers with approved projects who undergo the same security clearance as regular Census Bureau employees are granted access through Federal Statistical Research Data Centers.

6 In prior versions of this study, we used 2016 ACS data because they were the most recently available at that time.

7 We calculate this number using American FactFinder (AFF) Tables B98001 and B25001.

8 A parent can apply for the infant’s SSN at the hospital where the infant is born (enumeration at birth; see https://www.ssa.gov/policy/docs/ssb/v69n2/v69n2p55.html). Otherwise, applications for U.S.-born persons require an original or certified copy of a birth record (birth certificate, U.S. hospital record, or religious record before age 5, including the age), which the SSA verifies with the issuing agency, or a U.S. passport. Foreign-born U.S. citizen applications require a certified report of birth, consular report of birth abroad, U.S. passport, certificate of citizenship, or certificate of naturalization. Noncitizen applications require a lawful permanent resident card, machine-readable immigrant visa, arrival/departure record or admission stamp in an unexpired foreign passport, or an employment authorization document. See https://www.ssa.gov/ssnumber/ss5doc.htm.

9 A detailed history of the SSN is available at https://www.ssa.gov/policy/docs/ssb/v69n2/v69n2p55.html (Exhibit 1). For some categories of persons, the citizenship verification requirements started a few years later, but all were in place by 1978.

10 See https://www.ssa.gov/ssnumber/ss5doc.htm.

11 The Person Identification Validation System uses probabilistic matching to assign PIKs to each person. Each data set is matched to reference files, including the Numident and addresses from other federal files. See Wagner and Layne (2014) for details about the matching procedure.

12 The higher share of PIKs in the ACS compared with the 2010 census may reflect the fact that the 2010 census had proxy responses and the ACS does not. As noted earlier, proxy responses have very low PIK rates.

13 We classify U.S.-born persons with missing Numident citizenship as AR citizens in this analysis. We treat foreign-born persons with no ITIN or Numident citizenship as having missing AR citizenship, just as we do for persons who cannot be linked to ARs.

14 We cannot determine with certainty whether the administrative data or the ACS answer is correct. Unlike survey responses, the administrative data are verified at the time of application. The administrative data may not be fully updated when a person is naturalized, however. Brown et al. (2018) presented evidence suggesting that administrative data are correct most of the time when the two sources disagree. We thus classify an inconsistent response as problematic.

15 This method was initially developed to study the extent to which the gender or racial wage gaps are due to different distributions of characteristics associated with wages by gender or race (explained variation) versus differing behavior across gender or race for a given set of characteristics (unexplained variation). The unexplained variation is usually attributed to discrimination, but it captures any effects of differences in unobserved variables.

16 The reference person is self-identified as an individual who is responsible for the majority of the household mortgage or rent. Frequently, the reference person responds for the whole household.

17 High-quality linkage is defined as being linked in the first four passes of a module using address as well as name, date of birth, and gender. Lower-quality links are those made in all other passes. Layne et al. (2014) showed that the false match rate is much lower in the first four passes than for other linkage attempts. The first pass uses household street address, and later passes use increasingly higher levels of geography. The first four passes rely less on date of birth than the other passes or the other modules. Thus, any correlation between this variable and ACS–AR age disagreement due to the use of date of birth in the linkage should be negative. As a robustness exercise, we use the first pass using household street address, the linkage attempt least dependent on age as the proxy for better record linkage. The results are very close to those in Table 2.

18 Singer et al. (1992) reported that item nonresponse for the SSN question in the Simplified Questionnaire Test increased by person number in the household roster. In the 2000 Census Social Security Number, Privacy Attitudes, and Notification Experiment, Guarino et al. (2001) found that SSN item nonresponse was higher for person 2 than for person 1, and for persons 3–6 than for person 2. Brudvig (2003) found that SSN validation rates decreased with person number.

19 Although there is a Spanish version of the questionnaire and interviewers can provide support in more than 30 languages, comprehension could still be lower among those speaking English less well. McGovern (2004) found that households speaking a foreign language at home have higher item allocation rates for citizenship and related questions on the ACS.

20 An answer may be edited when it conflicts with other information provided about the person of interest. As a robustness check, we perform the Blinder-Oaxaca decomposition with a different definition of problematic response in which edited responses are considered problematic only if they disagree with the person’s AR. The results are qualitatively similar to those in Table 3.

21 We multiply the coefficients by 100 so that the results are expressed in percentages.

22 We exclude Puerto Rico.

23 Not only is the ACS questionnaire much longer than the 2010 census questionnaire, but it contains several potentially sensitive questions, such as income and public assistance receipt.

24 If all-citizen households are more likely to self-respond because of the presence of the citizenship question in the ACS, that will upwardly bias our estimate of the citizenship question unit self-response effect.

25 We multiply the coefficients by 100 so that the results are expressed in percentages.

26 Their model is used to produce the low response score in the Census Planning Database.

27 We do not include median house value because it is measured differently in the ACS over time and thus cannot be used in the same way for 2010 and 2017 estimates. This is the least powerful predictor in the Erdman and Bates model.

28 For 2017 **X**s, we use 2012–2016 five-year ACS tract population density.

29 Brown et al. (2018) reported that when the 2011–2015 five-year ACS is used to sort census tracts by the noncitizen share, this share is 0.0 % to 0.6 % in the bottom decile and 25.5 % to 100 % in the top decile, showing variation in noncitizen concentration by census tract.

30 The ACS does not have community outreach programs.

31 This is only part of the total change in unexplained variation between 2010 and 2017. The model coefficients could also change over this period, but they are unobserved in 2017.

32 Brown et al. (2019) comprises the entire replication archive containing all source code used for this analysis and is available at: 10.5281/zenodo.3275667.

33 Decennial census enumerators are less experienced than ACS field representatives, so they may be less able to get respondents to cooperate on the citizenship question.

34 See the online appendix for a discussion of robustness exercises using alternative sets of explanatory variables. These tests produce somewhat lower effects, estimating a 6.3 to 7.2 percentage point drop in 2010 in self-response rates due to the addition of a citizenship question.
