# Supplementary material for: Predicting the Effect of Adding a Citizenship Question to the 2020 Census
Source: Demography. 2019 Jul 17;56(4):1173–94. doi: 10.1007/s13524-019-00803-4 (PMC6667411; doi:10.1007/s13524-019-00803-4)
Supplement: Supplementary file 1 — (DOCX 17 kb) [file 13524_2019_803_MOESM1_ESM.docx]

ONLINE APPENDIX: BLINDER-OAXACA UNIT RESPONSE ROBUSTNESS

The differential ACS-Census self-response Blinder-Oaxaca decomposition results could potentially be sensitive to the choice of **X** characteristics used in the models. Though researchers typically select regressors for the decompositions based on theoretical considerations, an alternative would be to control for the variation in the ACS survey as a whole. As robustness checks we use two common machine learning methods for fitting models when a large number of variables are available.

In columns 1–3 of Table A.1, we implement different versions of the lasso procedure (see Friedman, Hastie, and Tibshirani, 2010). Specification 1 selects the regressors via the EBIC information criterion, specification 2 employs a cross-validation method, and specification 3 employs a standard lasso using the AIC information criterion. We consider 227 variables from across the entire 2010 ACS questionnaire in addition to the 39 used in Table 3;^[[1]](#footnote-1)^ 149 variables are selected by the lasso procedure in specification 1 and 157 in specifications 2 and 3. We obtain these lists of regressors from estimating an unweighted model of response on all-citizen households only.^[[2]](#footnote-2)^ We then use the resulting list of regressors in Blinder-Oaxaca decompositions like those in Table 4. For tractability, we exclude categorical variables with more than 20 distinct values (county of residence, industry, and moving date indicator variables). We also allow the lasso procedure to select indicator variables independently rather than requiring it to select all potential indicator variables for a given categorical variable. This means that unlike in Table 4, the Blinder-Oaxaca decompositions here do not normalize categorical variables. The only variable used in Table 4 that is not selected by the lasso is a single age category in specification 1.

Specifications 4–6 employ a list of regressors generated by principal component analysis (PCA; see Jackson 2003) using the same variables considered in the lasso regressions. All PCA regressors are generated from a standard PCA analysis applied to all households, not employing survey weights. Specification 4 uses the top 20 PCA components as the regressors in the Blinder-Oaxaca decomposition, specification 5 uses the top 50 components, and specification 6 uses the top 100 components.

Table A.1 shows that the unexplained component when using lasso model selection is 6.3–6.4 percentage points, and it is 7.0–7.2 when using PCA. These are smaller than the 8.8 percentage point unexplained component in Table 4, but they are still sizeable.

**REFERENCES**

Friedman, J., Hastie, T., and Tibshirani, R. (2010). “Regularization Paths for Generalized Linear Models via Coordinate Descent,” *Journal of Statistical Software*, *33*, 1–22. Retrieved from <https://www.jstatsoft.org/article/view/v033i01>

Jackson, J. E. (2003). *A User’s Guide to Principal Components*. New York: Wiley.

**Table A.1. Blinder-Oaxaca Decomposition of the Differences in 2010 ACS to 2010 Census Self-Response Rates by Household Citizenship Type. Robustness Tests with Alternative Sets of Controls**

|  | (1) | (2) | (3) | (4) | (5) | (6) |
| --- | --- | --- | --- | --- | --- | --- |
| Difference-in-differences | -11.91 | -11.91 | -11.91 | -11.91 | -11.91 | -11.91 |
|  | (0.0712) | (0.103) | (0.103) | (0.0692) | (0.0692) | (0.0692) |
| Explained | -5.580 | -5.540 | -5.540 | -4.762 | -4.956 | -4.836 |
|  | (0.0940) | (0.0895) | (0.0895) | (0.0694) | (0.0743) | (0.0814) |
| Unexplained | -6.333 | -6.373 | -6.373 | -7.151 | -6.957 | -7.077 |
|  | (0.130) | (0.140) | (0.140) | (0.107) | (0.105) | (0.112) |
| Number of regressors | 149 | 157 | 157 | 20 | 50 | 100 |
|  |  |  |  |  |  |  |

Source: ACS 1-year file, Census Unedited File (CUF), Census Numident, and ITINs, 2010. The Disclosure Review Board release number is DRB-B0035-CED-20190322.

Notes: Only NRFU-eligible housing units are included. 2010 CUF self-response is non-blank response to the first mailing, and ACS self-response is mail response. The standard errors are in parentheses, and they are bootstrapped using 80 ACS replicate weights. The number of observations is 1,418,000.

1. We exclude variables directly related to citizenship: place of birth, number of years in the U.S. (which is asked if the person selects a foreign country for place of birth), citizenship, and naturalization year. Those who select the U.S. as the place of birth are not asked the citizenship question. [↑](#footnote-ref-1)
2. We focus on all-citizen households here, because we are looking for regressors that can explain self-response behavior independent of the citizenship question. [↑](#footnote-ref-2)
